# Dataset on the variability of the light field in coastal waters

**DOI:** 10.1016/j.dib.2024.110923

**Published:** 2024-09-12

**Authors:** Stella Patricia Betancur-Turizo, Adan Mejia-Trejo, Yerinelys Santos-Barrera, Tatiana Marin-Amado, Erica Paola Zapata-Valezuela, Joaquín Pablo Rivero-Hernández, Rosana del Pilar Adames-Prada

**Affiliations:** a Centro de Investigaciones Oceanográficas e Hidrográficas del Caribe (CIOH), Cartagena 130001, Colombia; b Instituto de Investigaciones Oceanológicas, Universidad Autónoma de Baja California, km. 103 carretera Tijuana-Ensenada, Ensenada, Baja California 22860, Mexico; c Escuela Naval de Cadetes “Almirante Padilla”, Isla Naval Manzanillo, Cartagena de Indias 130001, Colombia

**Keywords:** Optical properties, Light field in the coastal water, Cartagena bay

## Abstract

The authors present data on both inherent and apparent optical properties, CTD profiles for the southwestern area of the Bay of Cartagena (Colombia) along a transect of seven stations. The data were collected during the dry and wet seasons of 2022. Optical properties include the depth of the Secchi disk as well as the absorption coefficients of particulate organic matter (ap) and chromophoric dissolved organic matter (aCDOM), together with analyses of total suspended solids (TSS) and turbidity in terms of nephelometric units (NTU). The dataset encompasses several types of data files on the light field in water, which is suitable for the development of water quality indices, the study of optically complex systems occupied by strategic marine ecosystems, the input of the calibration and validation processes of satellite algorithms as well as coastal zone management and administration.

Specifications Table

**Table:** 

Subject	*Hydrology and Water quality*
Specific subject area	*Evaluation of the light field in water.*
Type of data	Table, Raw
Data collection	*How the data were collected:* *Physical data were collected using RBR CTD; Water transparency was measured using an oceanographic Secchi disk (white disk of 30cm diameter) and those measurements were used to determine the vertical diffuse attenuation coefficient for the downward irradiance (Kd).* *Water samples were collected using* 5 L *Niskin bottles.* *Laboratory analyses of the collected water samples were performed using a spectrophotometer without integrating sphere (Varian Cary 100) to determine the absorption coefficients of particulate and dissolved matter.* *Matlab (version R2021a) routines were used for physical and bio-optical data processing.*
Data source location	*Institution: Centro de Investigaciones Oceanográficas e Hidrográficas del Caribe (CIOH).* *City/Town/Region: Cartagena de Indias/Bolívar* *Country: Colombia* *Location of collected data: Data was collected in seven monitoring stations (S01 to S07) at two depths (Surface: 1m; bottom: 10–30m), in the southern part of the Cartagena bay during two sampling campaigns according to different seasonal periods:* wet *and dry seasons.*
Data accessibility	Repository name: Catálogo de Metadatos del Cecoldo Data identification number: bc3ac94a-f23d-4b20–8c73–70ea6b2980fd Direct URL to data: https://cecoldo.dimar.mil.co/geonetwork/srv/spa/catalog.search#/metadata/bc3ac94a-f23d-4b20–8c73–70ea6b2980fd Instructions for accessing these data: …
Related research article	*None*

## 1 Value of the Data

• The data set of inherent optical properties of absorption and apparent properties have a potential for use in the field of water quality assessment, particularly in assessing light quality in coastal water.
• Determine the relationship between apparent and inherent optical properties of estuarine systems.
• The data set can be incorporated into national or regional assessments to contribute to coastal zone conservation and management programs.

## 2 Background

In the last decade, the nations of the world have set out to address the planet's environmental problems. Governments are incorporating innovative strategies into their national plans to help them understand the variability of coastal systems, where diverse economic, social and environmental activities converge and require quick decisions based on nature and its ecosystems. The idea is to choose a series of variables that, in their combination, can help us to describe the dynamics and variability associated with some processes. For example, the description of the variability of the in-water light field, can be based on Inherent Optical Properties (IOPs), and its implementation can be easily incorporated by most the centers for environmental monitoring, with existing resources. These properties modify the light field in the water column, according to their optical constituents (particulate and dissolved), so if we evaluate the contribution of each one, we can know if the conditions change. The objective of this dataset is to describe the variability of the light field in water, considering the variability of each component as well as the factors that make them vary individually, as a whole and to describe scenarios.

## 3 Data Description

**Fig. 1:**
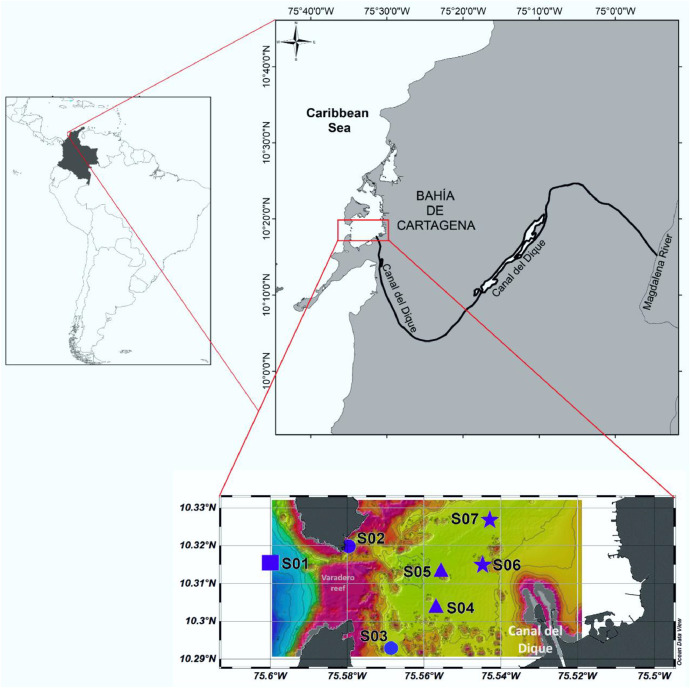
Locations of stations in the southwestern part of the Bahía de Cartagena, according to highest influence of turbid river water (blue stars), stations with intermediate influence between turbid river water and oceanic characteristics (blue triangles), stations with less influence of turbid river water (blue circles) and station with oceanic characteristics (blue square). (For interpretation of the references to color in this figure legend, the reader is referred to the web version of this article.)

The data set described in this article provides valuable information on the dynamics of water quality in the southwestern part of the Bahía de Cartagena along a transect of stations distributed in: stations with highest influence of turbid river water (S06 and S07), stations with intermediate influence between turbid river water and oceanic characteristics (S04 and S05), stations with less influence of turbid river water (S02 and S03) and stations with oceanic characteristics (S01) (Fig. 1). This dataset integrates the determination for the first time of the absorption coefficients of particulate and dissolved organic matter in surface (1 m) and bottom (10–30 m) samples, during two seasonal periods: dry season (February 18, 2022) and wet season (June 23, 2022).

The data set contains five types of data files: co_dimarcioh_*_Discrete_samples, co_dimarcioh_*_ ctd_DrySeason_0_31m, co_dimarcioh_*_ ctd_WetSeason_0_54m, co_dimarcioh_*_ctd_rbr_RawData and co_dimarcioh_*_Absorption_spectra. The measurements comprising each data file have been organized and identified according to the name of each column heading, corresponding unit and a brief description (Table 1). The data files co_dimarcioh_*_ctd_rbr_RawData correspond to different folders generated for the instrument software (RUSKIN) and are not included in Table 1.

**Table 1:** Description of the dataset according to type of data files.

Types of information	Column name	Unit	Description
co_dimarcioh_20220218_20220623_HydrologWaterQuality_Discrete_samples	Season	ID	Unique identifier for each seasonal period
	Station	ID	Unique identifier for each station of sampling
	Date:time	UTC-5	Date and time of sample or data collection in Coordinated Universal Time - 5 h (UTC-5). Datetime displayed as YYYY-MM-DDTHH:MM:SS
	Longitude	degrees_east	Longitude in decimal degrees obtained with GPSMAP 78S Garmin
	Latitude	degrees_north	Latitude in decimal degrees obtained with GPSMAP 78S Garmin
	Depth of sample collected	m	Total water depth in meters
	Zone	ID	Unique identifier for sampling area
	Depth station	m	Depth at which water sample is collected
	Temperature	°C	Temperature of the water in degrees Celsius
	Salinity	PSU	Salinity of the water in practical salinity unit (PSU)
	Z_SD_	m	Depth of the Secchi disk
	Kd model	m^-1^	Vertical diffuse attenuation coefficient for the downward irradiance, calculated from the model developed by Castillo-Ramírez et al. (2020)
	TSS	mg/L	Total Suspended Solids (TSS)
	Turbidity	NTU	Described as the opaqueness of a fluid due to the presence of suspended solids and is measured in terms of nephelometric turbidity units (NTU)
	a_CDOM_ (440)	m^-1^	Absorption coefficients of Chromophoric Dissolved Organic Matter at 440 nm
	ap (440)	m^-1^	Absorption coefficients for particulate absorption at 440 nm = Absorption coefficients of phytoplankton at 440 nm + Non-Algal Particles at 440 nm
	aTOTAL (440)	m^-1^	Total absorption coefficients for particulate and dissolved absorption at 440 nm = Absorption coefficients of CDOM at 440 nm + ap at 440 nm
	% ap (440)	%	Relative contribution of particulate absorption, to total non-water absorption at 440 nm
	% aCDOM (440)	%	Relative contribution of CDOM to total non-water absorption at 440 nm
	a_CDOM_ (443)	m^-1^	Absorption coefficients of Chromophoric Dissolved Organic Matter at 443 nm
	ap (443)	m^-1^	Absorption coefficients for particulate absorption at 443 nm = Absorption coefficients of phytoplankton at 443 nm + Non-Algal Particles at 443 nm
	aTOTAL (443)	m^-1^	Total absorption coefficients for particulate and dissolved absorption at 443 nm = Absorption coefficients of CDOM at 443 nm + ap at 443 nm
	% ap (443)	%	Relative contribution of particulate absorption, to total non-water absorption at 443 nm
	% aCDOM (443)	%	Relative contribution of CDOM to total non-water absorption at 443 nm
	a_CDOM_ (λ)	m^-1^	Additional data of absorption coefficients of Chromophoric Dissolved Organic Matter at 715, 676, 650, 630, 555, 510, 488 and 412 nm
	ap (λ)	m^-1^	Additional data of absorption coefficients for particulate absorption at 715, 676, 650, 630, 555, 510, 488 and 412 nm
*co_dimarcioh_20220218_HydrologWaterQuality_ctd_DrySeason_0_31m*	Time	UTC-5	Date and time of sample or data collection in Coordinated Universal Time - 5 h (UTC-5). Datetime displayed as YYYY-MM-DDTHH:MM:SS
	Longitude	degrees_east	Longitude in decimal degrees obtained with GPSMAP 78S Garmin
	Latitude	degrees_north	Latitude in decimal degrees obtained with GPSMAP 78S Garmin
	Station	ID	Unique identifier for each station of sampling
	Bot. Depth	m	Total water depth in meters
	Depth	m	Depth of each data taken by the CTD in Dry season
	Temperature	°C	Temperature of the water in degrees Celsius measured by CTD in Dry season
	Salinity	PSU	Salinity of the water in practical salinity unit (PSU) measured by CTD in Dry season
	Density	kg/m^3^	Density of the water in Kg/m3 measured by CTD in Dry season

*co_dimarcioh_20220623_HydrologWaterQuality_ctd_*Wet*Season_0_54m*	Time	UTC-5	Date and time of sample or data collection in Coordinated Universal Time - 5 h (UTC-5). Datetime displayed as YYYY-MM-DDTHH:MM:SS
	Longitude	degrees_east	Longitude in decimal degrees obtained with GPSMAP 78S Garmin
	Latitude	degrees_north	Latitude in decimal degrees obtained with GPSMAP 78S Garmin
	Station	ID	Unique identifier for each station of sampling
	Bot. Depth	m	Total water depth in meters
	Depth	m	Depth of each data taken by the CTD in Wet season
	Temperature	°C	Temperature of the water in degrees Celsius measured by CTD in Wet season
	Salinity	PSU	Salinity of the water in practical salinity unit (PSU) measured by CTD in Wet season
	Density	Kg/m3	Density of the water in Kg/m3 measured by CTD in Wet season

co_dimarcioh_20220218_20220623_yHydrologWaterQuality_Absorption_spectra	Wavelength (nm)	nm	Wavelength scanned every one nm from 800 nm to 400 nm
	S01	ID	Station with oceanic characteristics
	S02	ID	Station with less influence of turbid river water
	S03	ID	Station with less influence of turbid river water
	S04	ID	Station of intermediate influence between turbid river water and oceanic characteristics
	S05	ID	Station of intermediate influence between turbid river water and oceanic characteristics
	S06	ID	Station with greater influence of turbid river water
	S07	ID	Station with greater influence of turbid river water

The co_dimarcioh_*_Discrete_samples data file has three spreadsheets with field information taken at depth samples, seasonal periods and basic statistics. The first spreadsheet (Surface) provides the name and coordinates of the stations, date, time, depth, physical–chemical and bio-optical variables for Wet and Dry seasons, taken at a depth of one meter. The second spreadsheet (Bottom) contains the same data structure described in the surface sheet, but for samples collected in the deep layer (10–30 m). In the third spreadsheet (Statistic), you can find the calculations applied to the selected data set in order to identify significant differences between the Wet and Dry seasonal periods. Column definitions of the *Discrete_samples* data file are provided in Table 1.

**Fig. 2:**
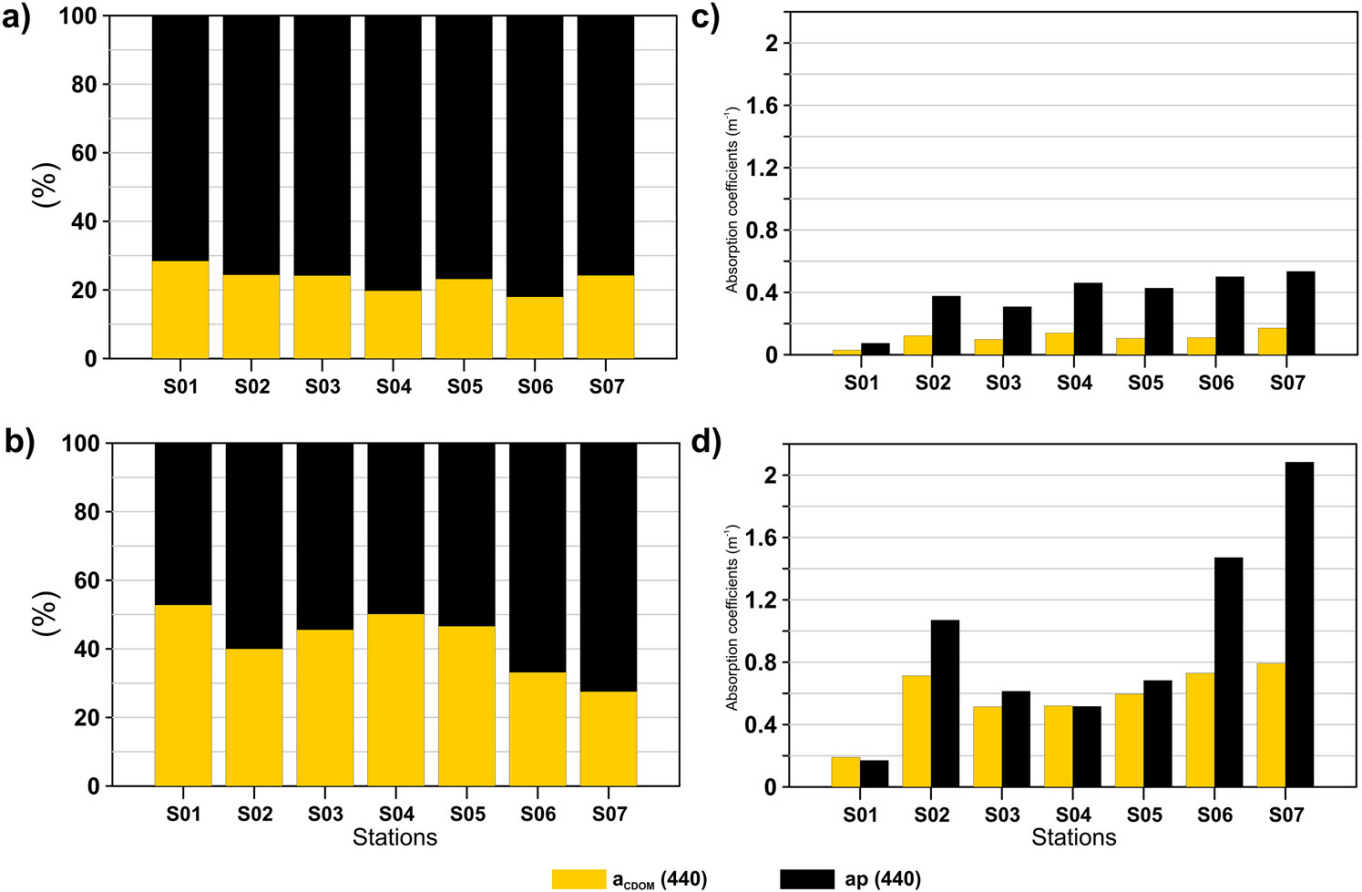
Relative contribution of aCDOM (440) and ap (440) to total non-water absorption of surface samples, during a) Dry season and b) Wet season. Absorption coefficients of aCDOM (440) and ap (440) of surface samples, during c) Dry season and d) Wet season.

The distribution of the absorption budget at 440 nm (Fig. 2) indicates that the particulate material (ap) is the component that dominates for most observations in the dataset, especially during Dry season, where ap (440) values were observed above 0.6 m^−1^(Fig. 2c), while during Wet season ap (440) values were below 0.4 m^−1^(Fig. 2d), except station S01. A Wilcoxon rank sum test was applied for the model Kd (m^−1^), TSS (mg/L), Turbidity (NTU), aCDOM (440) (m^−1^) and ap (440) (m^−1^). The results indicated that only aCDOM (440) (m^−1^) showed significant differences between the Wet and Dry seasons.

This data matrix also contains the absorption coefficients of aCDOM and ap for different wavelengths (715, 676, 650, 630, 555, 510, 488 and 412 nm), which can be used by researchers working on the validation of satellite algorithms for variables associated with coastal systems.

**Fig. 3:**
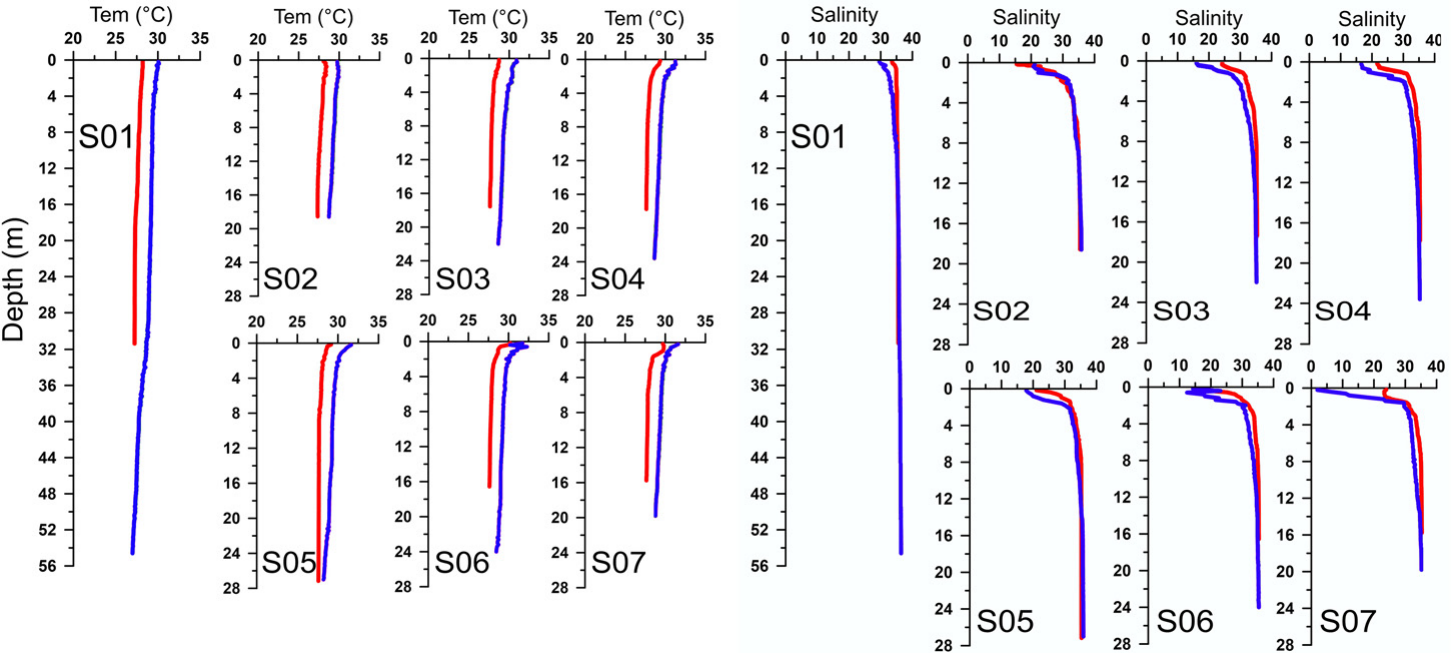
Temperature (°C) and salinity (psu) profiles, for Dry (red line) and Wet (blue line) season. (For interpretation of the references to color in this figure legend, the reader is referred to the web version of this article.)

In co_dimarcioh_*_ ctd_DrySeason_0_31 m and co_dimarcioh_*_ ctd_Wet*Season_0_*54 m, co_dimarcioh_*_ctd_rbr_RawData data files, you will find CTD profiles for each seasonal period (Wet and Dry). Each CTD file has seven spreadsheets, one for each monitoring station. Each sheet has temperature, salinity and density profiles with their respective coordinates, date and time of sampling defined in Table 1. This data set shows (Fig. 3) differences between the surface and bottom layers, with the highest temperatures and lowest salinities at the surface level, and the lowest temperatures and highest salinities at bottom layer. Between climatic periods, it is observed that the high temperatures and lowest salinities correspond to the Wet season, while the Dry season shows the lowest temperature values, and the highest salinity values.

**Fig. 4:**
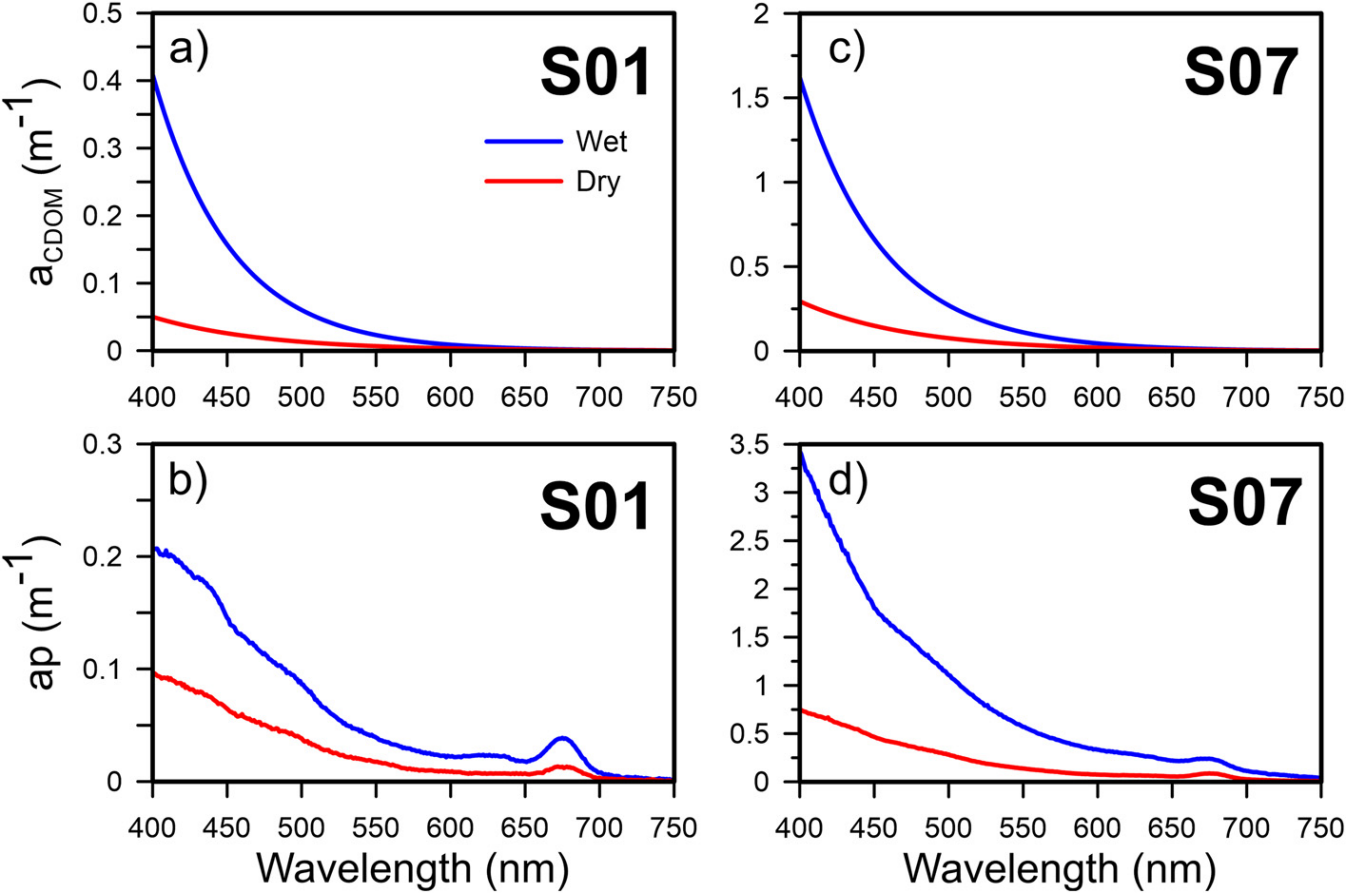
Absorption spectra of (a and c) aCDOM (m^−1^) fitted, and (b and d) ap (m^−1^) under oceanic conditions (station S01) and conditions of high influence of turbid river water (station S07). The red lines correspond to the Dry season and the blue lines to the Wet season. (For interpretation of the references to color in this figure legend, the reader is referred to the web version of this article.)

The co_dimarcioh_*_Absorption_spectra data file contains two files with absorption spectra of each coefficient analyzed in each seasonal period. The *Absorption spectra of ap and aCDOM_Wet* excel file has four spreadsheets for Rainy season, the first and second have the ap (λ) spectra for each of the seven stations (S01 to S07) surface and bottom, respectively (Fig. 4 b y d). The third and fourth spreadsheets, have the aCDOM (λ) spectra unfitted for each of the seven stations (S01 to S07) surface and bottom, respectively, but in this spreadsheet you can find the spectra of aCDOM fitted (Fig. 4a y c). A simple exponential model (*y*=Ae^((-S*λ)) was calculated using the wavelength range between 350 and 500 nm and the application of the nonlinear least squares method were used for its determination. This same data structure is in *Absorption spectra of ap and aCDOM_Dry* excel file, but for samples collected in the Dry season.

Finally, the co_dimarcioh_*_ctd_rbr_RawData data set, contains two folders with five types of data files generated by the instrument software (Ruskin 2.20.9.202403192006). Each file name consists of the instrument series, the date of data download, the time of data download and the extensions corresponds to:

• Raw binary (*.bin)
• Ruskin (*.rsk)
• Text (*.zip)
• Microsoft Excel (*.xlsx)
• OceanData View spreadsheet (*.odv)

## 4 Experimental Design, Materials and Methods

### 4.1 Sampling collection

Two samplings were developed for the seven monitoring stations (S01 to S07) in Dry (February 18 2022) and rainy seasons (June 23, 2022) (Fig. 1). Samples were taken at two depths (Surface: 1 m and bottom: 10–30 m) and collected using 5 L Niskin bottles for the determination of the variables analyzed in the laboratory. Water samples were collected directly from the Niskin bottle using pre-washed amber glass bottles. CDOM absorption was determined following the method described by Mitchell et al. [1]. The term absorption coefficient always refers to a single fixed wavelength (i.e. 440; 443; 412; 488; 510; 555; 630; 650; 676) They were stored under refrigeration (4 to 8 °C) until laboratory analysis. From the remaining volume, water samples were taken in 2000 mL plastic bottles for particulate matter determination, according to the protocol of Roesler et al. (2018) and 500 ml plastic bottles was used for turbidity and Total Suspended Solids (TSS) samples, according to the standard methods 23 edition [2].

*Physical data* were collected from the water column at all stations using a RBR CTD. The protocols defined by the manufacturer were followed for extraction, the data from the up and down cast have been combined to give the best possible measurement of the profile.

*Water transparency* was measured using an oceanographic Secchi disk (white disk of 30 cm diameter). The procedure applied for measuring this variable considered that the measurement can be affected by the human eye and therefore one person was assigned as responsible for this measurement. The differences associated with the angle were also considered, so the reading was made during the same time interval (between 9am and 3pm) mainly on sunny days, when it is assumed that there is more indirect light than diffuse light penetrating the water column [3]. When considering these aspects, it was assumed that errors were consistent (or constant) throughout the sampling process. Finally, Secchi disk measurements were used to determine the vertical diffuse attenuation coefficient for the downward irradiance (Kd).

### 4.2 Analytical methods

Once at the laboratory, 100 mL to 1000 mL of water was filtered through a negative filtration system, using Whatman GF/F filter (nominal pore size 0.7 µm and diameter of 25 mm) at low vacuum pressure (< 5 PSI), for the *analysis of the light absorption coefficient by particulate material* [4]. Each filter was carefully placed in Histopred capsules and stored in liquid nitrogen until spectrophotometric analysis. The filters stored for particulate matter were read, according to the protocol of Roesler et al. (2018), which contemplates the determination of the optical densities of the samples in a UV–vis spectrophotometer, in the absence of integrating sphere, in an optical scan between 400 nm to 800 nm. To obtain the particulate organic matter absorption coefficients of the samples analyzed, applying the following equation [4]:

(1) ap(λ)=ln(10)[0.679*(ODf(λ))∧1.2804]/(V/A)

where the units of filtrate volume (V) are given in m^3^, the effective filtration area (A) is in m^2^, the subscript 'p' represents the particulate components, and the factor [0.679 * (ODf (λ))^1.2804] represents the sample optical density (ODs (λ)) corrected by the amplification factor β for T-mode (Transmittance), which uses a spectrophotometer without an integrating sphere.

The *CDOM samples* were analyzed following the methodology of Mitchell et al. [1], which consisted of a filtering process of the sample through a 0.2 µm pore size membrane filter, previously washed with 10 % HCl and rinsed in ultrapure water. The previously treated filter was placed in a filtration system, through which a purging process was applied with ultrapure water and the sample itself. After this process, 75 ml of samples were passed through each purged filter to be poured into a quartz cylindrical cell of 10 cm optical path and read in a Varian Cary 100 UV–vis spectrophotometer, scanning between 400 nm and 800 nm. The absorption spectra obtained were processed, applying the Eq. (2), which allowed to determine the absorption coefficient of chromophoric dissolved organic matter [1]:

(2) aCDOM(λ)=(2.303/l)*((ODs(λ)−ODnull)−((ODb(λ)−ODnull)

where l is the cell path length (10 cm), ODs is the optical density of the sample, ODb is the optical density of the blank and ODnull is the optical density of the null point (600 nm).

For the determination of **Kd, the model** developed by Castillo-Ramírez et al. [5] was used ((3), (4)). This model proposes three depth-dependent equations of the Secchi disk (ZSD), as a representation of the range of optical diversity of the marine environment*,* as follows:

**Turbid water with ZSD < 2.20 m:**

(3) KdmodelC=1.16/((ZSD)∧0.62)

**Clear water with ZSD ≥ 5.37 m:**

(4) KdmodelC=0.62/((ZSD)∧0.72)

The *Total Suspended Solids (TSS) samples* were analyzed following the 2540 D Method, defined by Baird *et al*. [2], consisting of the determination of TSS using the gravimetry or weight difference technique. The procedure establishes the use of glass fiber filters (47 mm diameter and pore size ≤ 2 µm) previously heat-treated, at the time of analysis, each of these filters must be weighed together with a filter holder and the value recorded, then each one is taken using metallic tweezers and taken to the filtration equipment previously washed with deionized water. The collected sample is shaken with gentle circular movements for at least 15 s in order to guarantee homogeneity of our analyte, 300 ml are measured using glass graduated cylinders, the sample is passed through the filter until it is completely filled, rinsed with 100 ml of deionized water and dried for at least 1 h at 104 °C in an oven.

After an hour of drying, it must be cooled in a desiccator to balance the temperature, and then weighed. The cycle of drying, cooling, desiccating, and weighing is repeated until a constant weight is obtained or until the weight change is less than 4 % of the previous weight, or 0.5 mg, whichever is less.

To calculate the Total Suspended Solids (TSS), Eq. (5) is applied:

(5) mgTotalSuspendedSolids/L=((A−B)×1000)/samplevolume,mL

Where A is weight of filter + dried residue in mg; and B is weight of filter in mg.

As a quality control, a 50 mg/l solution of kaolin is prepared and subjected to the same procedure as a sample. The difference in weight should not exceed 4 %. Likewise, every 10 samples, one sample is taken at random and a duplicate is taken, the result of which should not exceed a difference in weight of 5 %.

The *measurement of turbidity in water* is measured using the nephelometry technique (radiation caused by particles) following the 2130 B Method defined by Baird et al. [2]. This method involves gently agitating the sample and taking an aliquot of at least 50 ml (it must be ensured that the sample is at room temperature). After this, the cell must be purged at least 2 times and the sample must be poured into the cell after the air bubbles have disappeared. The cell is dried on the outside so that no dirt or dust remains, and a wait for the reading to stabilize ensues until the data can be recorded. The turbidity should be read directly from the instrument display (Turbidity EZDO GONDO TUB-430 https://www.gondo.com.tw/products_detail/23.htm).

As quality control, a verification of the equipment should be made using reference material of 0, 20, 100, 200, 400 and 800 NTU. On the other hand, a duplicate should be read randomly and variability should not exceed 5 %.

## Limitations

None.

## Ethics Statement

The authors have read and follow the ethical requirements for publication in Data in Brief and confirm that the current work does not involve human subjects, animal experiments, or any data collected from social media platforms*.*

## CREDIT Author Statement

**Stella Patricia Betancur-Turizo**: Conceptualization, Methodology, Investigation, Writing - Original Draft, Writing - Review & Editing. **Adan Mejia-Trejo**: Software, Writing - Original Draft. **Yerinelys Santos-Barrera**: Investigation. **Tatiana Marin-Amado**: Investigation. **Erica Paola Zapata-Valezuela**: Investigation. **Joaquín Pablo Rivero-Hernández**: Methodology, Investigation. **Rosana del Pilar Adames Prada**: Project administration.

## Data Availability

Evaluation of the light field in water. (Original data) (Catálogo de Metadatos del Cecoldo).
